# Influence of a Nutrition Education Program on Parental Nutrition Knowledge, Dietary Habits, and Nutritional Status in Schoolchildren with Excess Weight

**DOI:** 10.3390/nu18040613

**Published:** 2026-02-13

**Authors:** Alexander Javier Iman Torres, Elí Guillermo López Padilla, Lesly Janela Baneo Shapiama, Carlos Antonio Li Loo Kung, Susy Karina Dávila Panduro, Jessy Patricia Vásquez Chumbe, Antonio Castillo-Paredes, Jose Jairo Narrea Vargas

**Affiliations:** 1 Departamento de Ciencia y Tecnología de Alimentos, Facultad de Industrias Alimentarias, Universidad Nacional de la Amazonía Peruana, Iquitos 16001, Peru; alexander.iman@unapiquitos.edu.pe (A.J.I.T.); lopezpadillaeli@gmail.com (E.G.L.P.); jbaneo.shapiama96@gmail.com (L.J.B.S.); jessy.vasquez@unapiquitos.edu.pe (J.P.V.C.); 2 Departamento de Ingenería de Alimentos, Facultad de Industrias Alimentarias, Universidad Nacional de la Amazonía Peruana, Iquitos 16001, Peru; carlos.li@unapiquitos.edu.pe; 3 Departamento de Ciencias Sociales, Facultad de Ciencias de la Educación y Humanidades, Universidad Nacional de la Amazonía Peruana, Iquitos 16000, Peru; susy.davila@unapiquitos.edu.pe; 4 Grupo AFySE, Investigación en Actividad Física y Salud Escolar, Escuela de Pedagogía en Educación Física, Facultad de Educación, Universidad de Las Américas, Santiago 8370040, Chile; acastillop85@gmail.com; 5 Grupo de Investigación en Nutrición, Metabolismo y Ejercicio, Facultad de Ciencias de la Salud, Carrera de Nutrición y Dietética, Universidad Científica del Sur, Lima 15067, Peru

**Keywords:** childhood obesity, nutrition education, dietary habits, health promotion

## Abstract

**Introduction:** Childhood obesity represents a major public health priority, and parent-focused educational interventions have been proposed as a strategy to improve dietary habits and prevent excess weight among schoolchildren. However, evidence regarding their impact on behavioral and anthropometric outcomes remains heterogeneous, particularly in Latin American settings. **Objective:** The aim of this study was to evaluate the influence of a parent-oriented nutrition education program on parental nutrition knowledge, dietary habits, and the nutritional status of schoolchildren with excess weight. **Methods:** A randomized controlled experimental study was conducted in a public educational institution in Peru. From a total population of 411 schoolchildren, a population-based screening was performed to identify those with excess weight; of the 198 children assessed, 64 met the inclusion criteria and were randomly assigned (1:1) to an intervention group or a control group. The educational program consisted of theoretical sessions, practical workshops, and home-based reinforcement over a three-month period. Outcomes included parental nutrition knowledge, children’s dietary habits, and body mass index-for-age Z-score according to sex. Longitudinal analyses were performed using linear mixed-effects regression models. **Results:** The intervention group showed a significant increase in parental nutrition knowledge compared with the control group (β = 5.65; 95% CI: 2.25–9.06; *p* = 0.002). No significant effects of the program were observed on dietary habits or BMI-for-age Z-score (*p* > 0.05). **Conclusions:** The nutrition education program significantly improved parental nutrition knowledge but did not lead to short-term changes in dietary habits or nutritional status among schoolchildren. These findings highlight the need for longer-term and multicomponent interventions to achieve meaningful behavioral and anthropometric changes.

## 1. Introduction

Overweight and childhood obesity constitute one of the most pressing public health challenges worldwide, with a rising prevalence among school-aged children and significant short- and long-term metabolic, cardiovascular, and psychosocial consequences [1]. Evidence indicates that dietary habits established during childhood tend to persist throughout the life course, underscoring the importance of early interventions aimed at promoting healthy eating behaviors and preventing excess weight gain [2].

In this context, school-based nutrition education programs have been widely implemented as strategies to improve nutritional knowledge and dietary behaviors in children. However, evidence regarding their impact on nutritional status remains heterogeneous [3]. Modeling studies and economic evaluations suggest that stand-alone nutrition education programs, implemented without structural or environmental modifications, often demonstrate limited effects on body mass index (BMI) and may not be cost-effective for the prevention of childhood obesity [4]. Consistently, systematic reviews and meta-analyses report that interventions based solely on nutrition education tend to produce modest or null improvements in anthropometric indicators when compared with multicomponent interventions [2,3].

Nevertheless, there is consistent evidence that well-structured educational interventions can lead to significant improvements in nutritional knowledge and diet quality, particularly when they are implemented over a sustained period and employ active learning methodologies. Recent quasi-experimental trials conducted in Middle Eastern school settings, such as a five-month school-based gardening intervention in Jordan, have demonstrated meaningful increases in nutritional knowledge, fruit and vegetable consumption, and overall diet quality, alongside reductions in BMI and BMI-for-age Z-scores, following interventions that integrate nutrition education with practical components such as school gardening activities [5]. Similarly, brief educational programs implemented in school and after-school settings among low-income populations in the United States have reported significant gains in nutritional knowledge; however, translating these improvements into sustained dietary behavior changes remains challenging [6].

Parental involvement has emerged as a key component in enhancing the effectiveness of nutrition interventions targeting children [7]. The literature indicates that parents directly influence food availability, feeding practices, and the home food environment, thereby shaping children’s dietary behaviors [8]. Interventions incorporating parent-focused components have been associated with improvements in nutritional knowledge, increased fruit and vegetable intake, and, in some cases, favorable changes in anthropometric indicators, particularly in vulnerable populations [5,9,10]. However, other studies suggest that although nutritional knowledge improves, behavioral changes may be limited when psychosocial, environmental, and family stress-related factors are not adequately addressed [6,9].

From a global perspective, the Food and Agriculture Organization of the United Nations (FAO), with the support of the World Health Organization (WHO), the United Nations Educational, Scientific and Cultural Organization (UNESCO), and the United Nations Children’s Fund (UNICEF), has promoted educational interventions aimed at improving dietary knowledge and practices among children [11]. Nevertheless, such efforts have primarily focused on large-scale population programs, and there remains a paucity of research evaluating nutrition education interventions in smaller, more specific contexts, such as schoolchildren with overweight. This gap limits the generation of context-specific and locally applicable evidence [3,12].

In South America, evidence on school-based nutrition education interventions remains limited and context-specific. Studies conducted in countries with socio-economic characteristics comparable to Peru, such as Chile and Paraguay, have reported improvements in nutritional knowledge and selected dietary behaviors following educational interventions among schoolchildren; however, their effects on anthropometric outcomes have been inconsistent and highly dependent on intervention design and family involvement [13,14]. Moreover, few studies in the region have specifically targeted children with overweight or incorporated parent-focused educational components, highlighting the need for further contextually relevant research.

Taken together, the available evidence suggests that nutrition education programs can consistently improve dietary knowledge and certain aspects of diet quality, but their impact on nutritional status depends on intervention design, duration, and, critically, the inclusion of the family environment. In this regard, it is pertinent to evaluate the influence of a parent-focused nutrition education program on dietary knowledge, dietary habits, and nutritional status among primary schoolchildren with overweight, in order to contribute to the existing body of evidence and to provide relevant insights for the development of more effective school-based intervention strategies.

## 2. Materials and Methods

### 2.1. Study Design

An experimental study with a control group and random allocation was conducted, following methodological principles for controlled trials in health and educational research and in accordance with the CONSORT guidelines for the reporting of randomized studies [15].

### 2.2. Participants

Participants were recruited from the Experimental UNAP Educational Institution, located in Peru, in the Loreto region, Maynas province, and San Juan Bautista district. The study population consisted of 411 primary schoolchildren enrolled during the year 2022.

### 2.3. Sample

The study was conducted in two stages. In the first stage, a population-based screening was carried out to identify schoolchildren with excess weight within the total population. Excess weight was identified through direct anthropometric assessment, including standardized measurements of body weight and height, followed by calculation of body mass index (BMI) and BMI-for-age Z-scores according to World Health Organization (WHO) growth standards (see Section 2.4.1). This approach is recommended when the condition of interest constitutes a specific inclusion criterion and its prevalence is unknown prior to recruitment [16,17].

The sample size for the screening phase was estimated using the finite population formula, assuming an expected proportion of 50%, a 95% confidence level (Z = 1.96), and a margin of error of 5%, resulting in an estimated sample of 198 schoolchildren. This conservative approach, based on maximum population variability (*p* = 0.5), is recommended when multiple outcomes are assessed and prior population estimates are unavailable [17]. Sample size calculation was performed using Epi Info software v7.2 (Centers for Disease Control and Prevention, Atlanta, GA, USA).

Subsequently, through written and verbal invitations directed to parents, the eligibility of schoolchildren included in the screening phase was assessed. After applying the inclusion and exclusion criteria, 134 schoolchildren were excluded. The eligible sample consisted of 64 schoolchildren with excess weight, defined as risk of overweight, overweight, or obesity (BMI-for-age Z-score > +1 SD according to WHO criteria).

In the second stage, schoolchildren identified with excess weight were included in a randomized experimental trial, with a 1:1 allocation to an experimental group (n = 32) and a control group (n = 32). Because the number of schoolchildren with excess weight was unknown prior to screening, the sample size for the trial was based on all cases identified during the screening phase.

Inclusion criteria were: schoolchildren of both sexes with active enrollment at the educational institution, whose parents voluntarily agreed to participate in the study, and who presented excess weight (risk of overweight, overweight, or obesity), defined as a value greater than +1 standard deviation (SD) according to the BMI-for-age indicator. Schoolchildren with physical and/or mental conditions that could compromise data collection were excluded.

**Figure 1:**
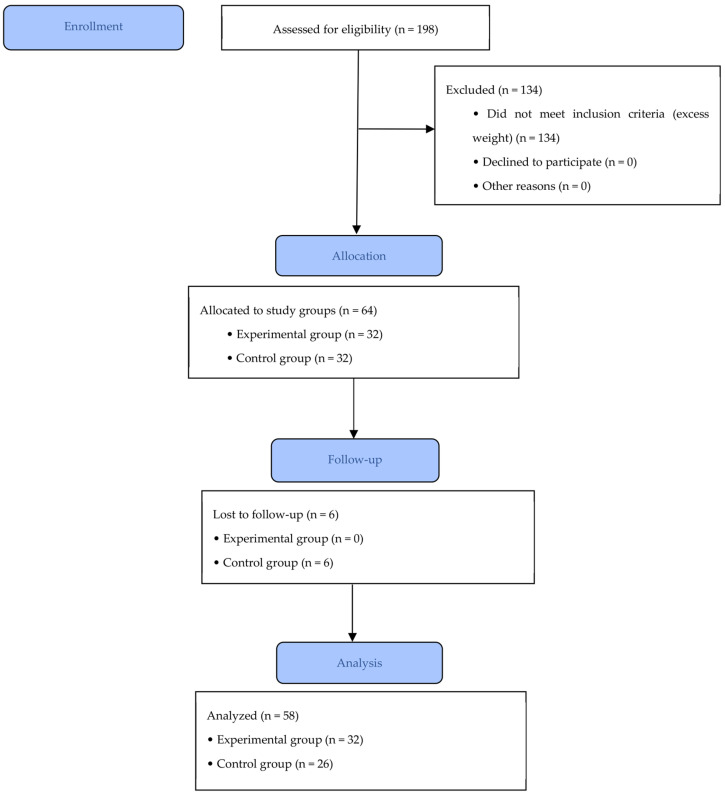
Flow of participants through the different stages of the study. The diagram shows the number of participants assessed for eligibility, excluded, allocated to the experimental and control groups, lost to follow-up, and included in the final analysis.

During the intervention period, a loss of six participants was recorded in the control group, attributed to parents’ absence during the nutritional knowledge assessment and failure to complete dietary habits or BMI-for-age evaluations. Consequently, the final analysis included 32 schoolchildren in the experimental group and 26 in the control group (Figure 1).

### 2.4. Intervention Procedures

Prior to study initiation, formal contact was established with the administration of the Experimental UNAP Educational Institution through a written request addressed to the corresponding educational authorities, in which the objectives, scope, and procedures of the study were outlined. Following institutional approval, coordination was carried out with the administrative team and responsible teachers to facilitate access for the research team and the implementation of the planned activities.

Subsequently, face-to-face informational meetings were held with parents or legal guardians of first- to sixth-grade schoolchildren. During these meetings, the study objectives, assessment procedures, the nature of the nutrition education intervention, potential benefits, and the voluntary nature of participation were explained in detail. After these meetings, written informed consent was obtained from parents, along with assent from the schoolchildren.

Once consent was obtained, baseline assessments were conducted, including anthropometric measurements of the schoolchildren, administration of the nutritional knowledge questionnaire to parents, and assessment of the schoolchildren’s dietary habits. Subsequently, the nutrition education program was implemented for parents in the experimental group.

Follow-up assessments were conducted three months after completion of the baseline evaluation, using the same instruments and procedures. All assessments and educational sessions were carried out between June and September 2022, after school hours (4:00–6:00 p.m.).

The control group did not receive any intervention during the follow-up period; however, for ethical reasons, the same nutrition education program was offered and delivered to this group after completion of the study.

#### 2.4.1. Anthropometric Nutritional Status

Anthropometric assessments were performed by researchers previously trained and standardized by a specialist in anthropometry, following current Peruvian regulations [16]. Body weight was measured with schoolchildren barefoot and wearing light clothing, using a digital Omron scale (model HBF-514C, Omron Healthcare Co., Ltd., Kyoto, Japan), with a precision of ±1% and automatic calibration. Height was measured using a stadiometer certified by the National Center for Food, Nutrition, and Healthy Living (CENAN, Lima, Peru), with a precision of 0.1 cm, ensuring correct anatomical positioning.

BMI was calculated as body weight divided by height in meters squared (kg/m^2^). Subsequently, BMI-for-age Z-scores adjusted for sex were calculated using WHO AnthroPlus software (v1.0.4., for children and adolescents aged 5–19 years), based on WHO growth standards. Anthropometric nutritional status was classified using standard deviations (SD): risk of overweight (>+1 SD to ≤+2 SD), overweight (>+2 SD to ≤+3 SD), and obesity (>+3 SD) [18,19,20].

#### 2.4.2. Nutritional Knowledge

Parental nutritional knowledge was assessed using a structured questionnaire administered before and three months after the nutrition education intervention. The instrument consisted of 11 items designed to evaluate knowledge related to healthy eating, child nutrition, and appropriate feeding practices during the school stage (the full questionnaire is provided as Supplementary Material File S1). Each correctly answered item was scored with 4 points, except for item No. 4, for which the maximum and minimum correct scores were 20 and 5 points, respectively, and item No. 9, with maximum and minimum scores of 24 and 6 points, respectively.

The questionnaire underwent content validation through expert judgment by four specialists in nutrition and food education. A pilot test was conducted in a sample of 100 parents with characteristics similar to the study population to assess internal consistency. Validity and reliability were determined using Aiken’s V and Cronbach’s alpha coefficients, yielding values of 0.83 and 0.87, respectively, which are considered adequate for intervention studies.

Based on total scores, nutritional knowledge was categorized as low (11–18 points), medium (19–35 points), and high (36–80 points).

#### 2.4.3. Dietary Habits

Dietary habits of the schoolchildren were assessed using a food frequency consumption questionnaire, administered before the intervention and three months after completion of the nutritional education intervention. The instrument was based on the questionnaire proposed by Esteban-Figuerola [21], which was adapted to the local context. The adapted version included eight food groups and a total of 69 items, assessed using a five-point Likert-type scale with the following response options: daily, 4–6 times per week, 1–3 times per week, 1–3 times per month, and never. The complete list of the 69-food frequency consumption questionnaire items is provided as Supplementary Material File S2.

Foods were classified as healthy or unhealthy according to the recommendations of the Peruvian Dietary Guidelines and the WHO. Foods recommended for regular consumption—such as fruits, vegetables, whole grains, legumes, unprocessed animal-source foods, and water—were classified as healthy, whereas foods with limited or non-recommended consumption—such as sugar-sweetened beverages, ultra-processed products, salty snacks, sweets, and foods high in added sugars, saturated fats, or sodium—were classified as unhealthy.

For food items considered within a ‘gray area’ (e.g., flavored yogurts, fortified breakfast cereals, and other mixed or processed products), classification was based on their nutritional profile and degree of processing, prioritizing the presence of added sugars, saturated fats, and sodium, in accordance with national and WHO guidelines. Items containing added sugars or classified as ultra-processed were scored as unhealthy.

Scoring was assigned according to the nature of the food item, using direct scoring for healthy foods and reverse scoring for unhealthy foods, such that higher total scores reflected less favorable dietary habits. Based on the total score obtained, dietary habits were classified as healthy (≤207 points) or unhealthy (≥208 points).

The questionnaire was validated by expert judgment from four specialists and pilot-tested in a population with characteristics similar to the study sample to evaluate internal consistency. Validity and reliability were assessed using Aiken’s V and Cronbach’s alpha coefficients, both yielding values of 0.92, which are considered adequate for intervention studies.

#### 2.4.4. Nutrition Education Program

The nutrition education program was designed following the AAMMEE methodology (Analysis, Attention, Motivation, Message, Exercise, and Evaluation) and was developed in accordance with the guidelines of the National Curriculum of Basic Education and the Primary Education Curriculum Program [22]. Educational content was structured by educational cycles: first cycle (1st–2nd grade), second cycle (3rd–4th grade), and third cycle (5th–6th grade), adapting educational messages to the cognitive level and comprehension capacity of schoolchildren.

The intervention was implemented after completion of baseline assessments, including anthropometric nutritional status of the schoolchildren, parental nutritional knowledge, and children’s dietary habits. The program was primarily targeted at parents in the experimental group, with active participation of their children, and was delivered over a three-month period.

The program consisted of two complementary components. The first component included theoretical educational sessions, aimed at strengthening parents’ nutritional knowledge and addressing topics such as healthy eating during the school stage, food groups, portion sizes, consumption frequency, and common misconceptions in child nutrition. The second component comprised practical demonstrative workshops, focused on the application of acquired knowledge, including the preparation of healthy school lunches and balanced meals using commonly available and low-cost foods.

Each educational session and demonstrative workshop lasted approximately 60 min and was conducted in person at the educational institution during after-school hours. In total, five face-to-face sessions were delivered, combining theoretical and practical activities throughout the intervention period.

As a reinforcement strategy, a multicomponent follow-up approach was implemented for parents in the experimental group. This included in-person home visits, conducted every 20 days, with an approximate duration of 20 min per family, during which educational content was reinforced, questions were addressed, and personalized recommendations were provided. During these visits, parents also received printed educational materials and a recipe booklet featuring healthy meal preparations adapted to the local context.

In addition, telephone counseling was provided as needed, and a WhatsApp group was created for parents in the experimental group. Through this platform, educational materials, motivational videos, and reinforcement messages were shared regularly. The group also enabled parents to share their progress, experiences, and challenges, fostering peer support and continuous engagement throughout the intervention period.

### 2.5. Ethical Considerations

The study was approved by the Ethics Committee of the National University of the Peruvian Amazon (approval code: No. 019-2023-CIEI-VRINV-UNAP), in accordance with the Declaration of Helsinki [23], as well as the Singapore Statement on Research Integrity, which establishes fundamental principles for responsible conduct of research [24]. Following institutional authorization from the school, written informed consent was obtained from parents or legal caregivers prior to any anthropometric assessment conducted during the screening phase, allowing access to their children’s data for the identification of excess body weight. In addition, assent was obtained from all schoolchildren. Data confidentiality was ensured, and all data were handled in compliance with Peruvian Law No. 29733 on Personal Data Protection and its regulations.

### 2.6. Statistical Analysis

Descriptive statistics were used to characterize the baseline sample, reporting means and standard deviations (SD) for continuous variables and absolute and relative frequencies for categorical variables. Normality of continuous variables was assessed using the Shapiro–Wilk test. Baseline comparability between experimental and control groups was evaluated using Student’s *t* tests for independent samples for numerical variables and chi-square tests or Monte Carlo simulations for categorical variables, as appropriate.

Pre- and post-intervention changes were analyzed using linear mixed-effects regression models, accounting for the longitudinal nature of the data. These models included a random intercept at the participant level to capture within-subject correlation of repeated measurements. Study group (experimental vs. control), time (post vs. pre), and the group × time interaction were modeled as fixed effects, with the interaction term serving as the primary parameter for estimating the effect of the nutrition education program.

Mixed-effects models were preferred over paired analyses or ANCOVA due to their greater flexibility in handling individual variability and their ability to provide unbiased estimates in the presence of missing data under the missing-at-random assumption. Robust standard errors were used to ensure valid statistical inference in the presence of potential deviations from homoscedasticity or normality. Models for dietary habits and anthropometric nutritional status were additionally adjusted for the child’s sex.

To illustrate individual changes in nutritional knowledge over time, paired raincloud plots were constructed using the ggplot2 package in R (v4.5.0). Statistical analyses were primarily performed using SPSS version 22.0 (IBM Corp., Armonk, NY, USA), and a *p*-value < 0.05 was considered statistically significant.

## 3. Results

The experimental (n = 32) and control (n = 26) groups presented comparable baseline characteristics at study entry (Table 1). No statistically significant differences were observed between groups in age, sex, school grade, parental educational level, or prior exposure to nutrition education (all *p* > 0.05).

Similarly, baseline scores for parental nutritional knowledge, schoolchildren’s dietary habits, and BMI-for-age Z-scores were comparable between groups, indicating adequate baseline equivalence prior to the intervention.

Table 2 presents the descriptive pre- and post-intervention changes in the evaluated outcomes by study group.

In the experimental group, the mean parental nutritional knowledge score increased markedly after the intervention, from 48.93 ± 7.40 to 53.43 ± 8.11 points (Δ = +4.5), whereas the control group showed a slight decrease in this indicator (Δ = −1.16).

In contrast, dietary habits did not show relevant changes over time. The intervention group exhibited a minimal change in mean score (Δ = −1.62), while the control group showed a slight increase (Δ = +1.89).

Regarding anthropometric nutritional status, both groups experienced modest and comparable reductions in BMI-for-age Z-scores over time. In the experimental group, the Z-score decreased by −0.24 units, whereas in the control group the reduction was −0.27, suggesting similar changes regardless of assignment to the educational program.

Linear mixed-effects regression models revealed a significant group × time interaction for parental nutritional knowledge, indicating a positive effect of the educational intervention. In the crude analysis, the intervention group showed an average increase of 5.65 points in nutritional knowledge scores compared with the control group (β = 5.65; SE = 1.7; 95% CI: 2.25–9.06; *p* = 0.002).

In contrast, no significant effects of the program were observed on schoolchildren’s dietary habits (95% CI: −3.32 to 10.33; *p* = 0.30) or on anthropometric nutritional status assessed by BMI-for-age Z-score (95% CI: −0.16 to 0.09; *p* = 0.60). Adjustment for sex did not modify the effect estimates for dietary habits or BMI-for-age (Table 3). Post-hoc comparisons based on estimated marginal means showed that parental nutritional knowledge increased markedly in the intervention group from pre- to post-intervention (+4.5 points), whereas the control group exhibited a slight decrease over time (−1.15 points). These findings indicate that the significant group × time interaction was primarily driven by the improvement observed in the intervention group rather than by changes in the control group (Table S1).

**Figure 2:**
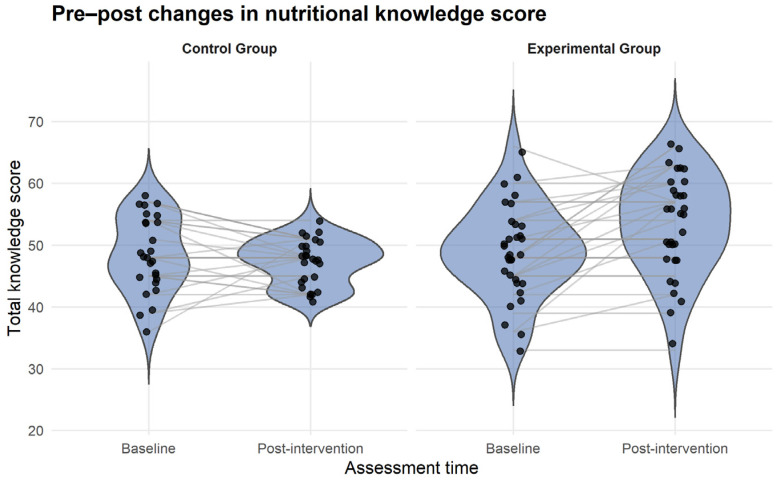
Individual changes in nutritional knowledge scores.

Individual changes in parental nutritional knowledge over time are illustrated in Figure 2.

## 4. Discussion

The present study evaluated the influence of a nutrition education program directed at parents on parental nutritional knowledge, dietary habits, and nutritional status of schoolchildren with excess weight, using an experimental design with a control group and random allocation. The main findings demonstrate a positive and statistically significant effect of the nutrition education program on parental nutritional knowledge, whereas no significant changes were observed in children’s dietary habits or anthropometric nutritional status during the follow-up period.

The significant increase in parental nutritional knowledge is consistent with previous evidence highlighting the central role of parents as key modulators of the child food environment. Recent studies have shown that educational interventions targeting caregivers consistently improve knowledge related to healthy eating, particularly when they focus on practical messages tailored to the family context [2,4,12]. Similarly, Kostecka et al. [25] reported substantial improvements in parental nutritional knowledge following prolonged interventions during the preschool years, reinforcing the relevance of family-based education as an initial strategy for change.

Despite the observed improvement in knowledge, the program did not produce statistically significant changes in children’s dietary habits. This finding aligns with reports by Yeh et al. [26] and Qian et al. [27], who indicate that increased knowledge does not necessarily translate into immediate behavioral change. The literature suggests that children’s eating behaviors are strongly influenced by structural, social, and environmental factors—such as food availability, school food environments, and established taste preferences—which may limit the impact of short-term educational interventions [6,8].

Moreover, it is important to acknowledge that children’s nutritional status results from the interaction of multiple determinants that extend beyond the variables assessed in the present study. Family-related factors—such as household feeding practices, parental involvement, food availability, and parental role modeling—as well as school-related factors, including food availability, institutional policies, and the broader obesogenic environment, may exert a substantial influence on childhood BMI. Previous studies conducted in South American settings, such as Chile and Paraguay, have shown that interventions simultaneously engaging the family, school, and community environments are more likely to achieve meaningful anthropometric changes than those focused solely on nutrition education [13,14,28]. In this context, the present findings suggest that while improvements in parental nutrition knowledge represent a necessary initial step, their impact on children’s body weight may be attenuated in the absence of sustained behavioral and structural changes within the environments in which children’s daily habits are formed.

### 4.1. Practical Implications

From an applied perspective, the results support the incorporation of parent-focused nutrition education programs as an effective strategy to improve nutritional knowledge in school settings. However, the findings also suggest that such interventions should be complemented by additional structural actions, such as modifications to the school food environment (including the implementation of healthy cafeterias and school meal services, as mandated by Peruvian law [29,30]), as well as family-based behavioral strategies, such as establishing household food rules (limiting sugar-sweetened beverages and ultra-processed snacks) and parental role modeling of healthy behaviors (visible consumption of fruits and vegetables and shared family meals). These combined approaches may better facilitate sustained improvements in children’s dietary habits and nutritional status.

### 4.2. Strengths and Limitations

This study has several strengths and limitations that should be considered when interpreting the findings. Among its strengths, the experimental design with a control group and random allocation enhances internal validity and supports causal inference regarding the effect of the nutrition education program. In addition, the two-stage population-based screening allowed for the identification and inclusion of schoolchildren with excess weight from a well-defined source population, improving the transparency of participant selection. The use of validated instruments with adequate reliability for assessing parental nutrition knowledge and children’s dietary habits, as well as standardized anthropometric measurements based on WHO growth references, further strengthens the methodological rigor of the study. Moreover, the inclusion of parents as the primary target of the intervention reflects a family-centered approach that is highly relevant for childhood nutrition research.

Despite these strengths, several limitations should be acknowledged. First, the relatively short follow-up period may have limited the detection of changes in dietary habits and anthropometric outcomes, as longer and more intensive educational interventions have been shown to be more effective in modifying complex and sustained eating behaviors [25]. Second, the use of self-reported questionnaires may have introduced social desirability bias.

Additionally, the sample size of the experimental trial was constrained by the number of schoolchildren with excess weight identified during the population screening phase, which was unknown a priori. This limitation, together with participant attrition during follow-up in the control group (n = 6), may have reduced the statistical power to detect between-group differences, particularly for anthropometric outcomes. Nevertheless, the initial random allocation was balanced between groups (n = 32 per group), which supports the internal validity of the comparisons.

### 4.3. Future Perspectives

Future research should consider longer-duration interventions with longitudinal follow-up and incorporate multicomponent strategies that integrate the school, family, and community environments. It would also be valuable to explore mediators of behavioral change, such as parental self-efficacy, availability of healthy foods, and family feeding practices, as well as to assess differential effects according to parental educational level and socioeconomic context.

## 5. Conclusions

The parent-directed nutrition education program achieved a positive and significant effect on nutritional knowledge, evidenced by greater improvements in the experimental group compared with the control group. This finding confirms that structured educational interventions, even of relatively short duration, can effectively strengthen caregivers’ nutritional knowledge, given their central role in household food-related decisions.

However, no significant changes were observed in children’s dietary habits or anthropometric nutritional status (BMI-for-age Z-score) during the follow-up period. These results suggest that, while knowledge is a necessary component, it is not sufficient on its own to induce sustained behavioral changes or short-term anthropometric improvements, particularly among schoolchildren with excess weight.

Overall, the findings underscore the need for comprehensive nutrition education programs that not only reinforce knowledge but also incorporate family-based behavioral strategies, environmental components, and longer follow-up periods to translate learning into effective changes in dietary habits and improvements in child nutritional status.

## Figures and Tables

**Table 1:** Baseline characteristics of schoolchildren and parents by study group.

Variable		Experimental Group (n = 32)	Control Group (n = 26)	*p*-Value
		Mean ± SD		
Age	years	9.51 ± 1.78	8.96 ± 1.79	0.92
Nutritional knowledge	points	48.93 ± 7.40	48.46 ± 6.1	0.50
Eating habits	points	205.96 ± 17.39	222.34 ± 22.22	0.19
BMI-for-age	Z-score	2.27 ± 0.83	2.21 ± 0.71	0.55
		n (%)		
Sex				
	Male	17 (53.1)	13 (50)	0.81
	Female	15 (46.9)	13 (50)	
School grade				
	First grade	4 (12.5)	6 (23.1)	0.79
	Second grade	5 (15.6)	6 (23.1)	
	Third grade	5 (15.6)	4 (15.4)	
	Fourth grade	6 (18.8)	3 (11.5)	
	Fifth grade	8 (25)	4 (15.4)	
	Sixth grade	4 (12.5)	3 (11.5)	
Parental educational level				
	Basic education	7 (21.8)	5 (19.2)	0.82
	Technical higher education	14 (43.8)	10 (38.5)	
	University education	11 (34.4)	11 (42.3)	
Parent received prior nutrition education				
	Yes	11 (34.4)	10 (38.5)	0.74
	No	21 (65.6)	16 (61.5)	

**Table 2:** Changes in nutritional knowledge, eating habits, and nutritional status by study group.

Variable	Group	Pre-Intervention	Post-Intervention	Δ
		Mean ± SD		
Nutritional knowledge (score)	Experimental	48.93 ± 7.40	53.43 ± 8.11	4.5
	Control	48.46 ± 6.10	47.30 ± 3.63	−1.16
Eating habits (score)	Experimental	205.96 ± 17.39	204.34 ± 17.62	−1.62
	Control	222.34 ± 22.22	224.23 ± 17.41	1.89
BMI-for-age (Z-score)	Experimental	2.27 ± 0.83	2.03 ± 0.79	−0.24
	Control	2.21 ± 0.70	1.94 ± 0.89	−0.27

Δ corresponds to the absolute change (post − pre).

**Table 3:** Effect of the nutrition education program on study outcomes (mixed-effects models).

Variable	Model	β	SE	95% CI	*p*-Value
Nutritional knowledge	Crude	5.65	1.7	2.25–9.06	0.002
Eating habits	Crude	3.50	3.4	−3.31–10.33	0.30
	Adjusted	3.50	3.4	−3.32–10.33	0.30
BMI-for-age	Crude	−0.03	0.06	−0.16–0.09	0.60
	Adjusted	−0.03	0.06	−0.16–0.09	0.60

β represents the effect of the group × time interaction (intervention vs. control) on the pre-post change. Models for dietary habits and BMI-for-age were adjusted for the child’s sex. SE: standard error; 95% CI: 95% confidence interval.

## Data Availability

The raw data supporting the conclusions of this article are not publicly available due to ethical and privacy restrictions related to the protection of participants’ personal data; however, they are available from the authors upon reasonable request.

## Supplementary Materials

The following supporting information can be downloaded at: https://www.mdpi.com/article/10.3390/nu18040613/s1, File S1: Parental Nutritional Knowledge Questionnaire; File S2: Food Frequency Questionnaire (FFQ)—69 Items; Table S1: Post-hoc pairwise comparisons of parental nutritional knowledge scores by group and time.

## Abbreviations

The following abbreviations are used in this manuscript:

BMI	Body mass index
FAO	Food and Agriculture Organization
WHO	World Health Organization
UNESCO	United Nations Educational, Scientific and Cultural Organization
UNICEF	United Nations Children’s Fund
AAMMEE	Analysis, Attention, Motivation, Message, Exercise, and Evaluation

## References

[1] Shamah-Levy T., Del Monte-Vega M.Y., Valenzuela-Bravo D.G., Morales-Ruan C., Moreno-Macías L., Galindo-Gómez C., Fajardo-Niquete I., Troconis-Cervera J. (2025). Health and Nutrition Interventions to Prevent Childhood Overweight and Obesity in Mexico and Latin America: A Systematic Review. Nutrients.

[2] Yoong S.L., Lum M., Wolfenden L., Jackson J., Barnes C., Hall A.E., McCrabb S., Pearson N., Lane C., Jones J.Z. (2023). Healthy eating interventions delivered in early childhood education and care settings for improving the diet of children aged six months to six years. Cochrane Database Syst. Rev..

[3] Pongutta S., Ajetunmobi O., Davey C., Ferguson E., Lin L. (2022). Impacts of School Nutrition Interventions on the Nutritional Status of School-Aged Children in Asia: A Systematic Review and Meta-Analysis. Nutrients.

[4] Kenney E.L., Poole M.K., McCulloch S.M., Barrett J.L., Tucker K., Ward Z.J., Gortmaker S.L. (2025). School-based nutrition education programs alone are not cost effective for preventing childhood obesity: A microsimulation study. Am. J. Clin. Nutr..

[5] Elsahoryi N.A., Alhaj O.A., Musharbash R., Milhem F., Al-Farah T., Al Jawaldeh A. (2025). A School-Based Five-Month Gardening Intervention Improves Vegetable Intake, BMI, and Nutrition Knowledge in Primary School Children: A Controlled Quasi-Experimental Trial. Nutrients.

[6] Nam K., Kulinna P.H., Pivovarova M., Kim J., Albaloul O. (2026). Evaluating a Nutrition Education Program’s Impact on Knowledge and Eating Behavior Among Low-Income Youth: A Social Cognitive Theory Perspective. J. Sch. Health.

[7] Varela E.G., Bonfiglio C., Zeldman J., Chavez A., Mobley A.R. (2025). Enhancing Nutrition Communication in Early Childhood Education Settings: Overcoming Challenges and Promoting Collaboration with Caregivers. Int. J. Environ. Res. Public Health.

[8] Krishnakumar P., Coccia C. (2023). Perceived Role of Asian Indian Fathers in Florida During Mealtimes: Factors to Consider for Their Involvement in Childhood Obesity Prevention. Fam. Community Health.

[9] Ling J., Chen S., Zhang N., Robbins L.B., Kerver J.M. (2024). Happy Family, Healthy Kids: A Healthy Eating and Stress Management Program in Low-Income Parent-Preschooler Dyads. Nurs. Res..

[10] Francis L.A., Nix R.L., BeLue R., Keller K.L., Kugler K.C., Rollins B.Y., Savage J.S. (2023). Designing a childhood obesity preventive intervention using the multiphase optimization strategy: The Healthy Bodies Project. Clin. Trials.

[11] Food and Agriculture Organization of the United Nations (FAO) (2020). School-Based Food and Nutrition Education: A White Paper on the Current State, Principles, Challenges and Recommendations for Low- and Middle-Income Countries.

[12] Hamulka J., Czarniecka-Skubina E., Gutkowska K., Drywień M.E., Jeruszka-Bielak M. (2023). Nutrition-Related Knowledge, Diet Quality, Lifestyle, and Body Composition of 7–12-Years-Old Polish Students: Study Protocol of National Educational Project Junior-Edu-Żywienie (JEŻ). Nutrients.

[13] González C.G., Domper A., Fonseca L., Lera L., Correa P., Zacarías I., Vio F. (2020). Aplicación y efectividad de un modelo educativo en hábitos saludables con entrega de fruta y programa de actividad física en escolares. Rev. Chil. Nutr..

[14] Villagra M., Meza E., Villalba D. (2020). Intervención Educativa-Nutricional sobre hábitos alimentarios aplicada a escolares de Asunción, Paraguay. Mem. Inst. Investig. Cienc. Salud..

[15] Schulz K.F., Altman D.G., Moher D. (2010). CONSORT 2010 Statement: Updated Guidelines for Reporting Parallel Group Randomized Trials. BMJ.

[16] Hulley S.B., Cummings S.R., Browner W.S., Grady D., Newman T.B. (2013). Designing Clinical Research.

[17] Lwanga S.K., Lemeshow S. (1991). Sample Size Determination in Health Studies: A Practical Manual.

[18] Ministerio de Salud (Perú) (2024). Resolución Ministerial N.° 034-2024-MINSA: Guía Técnica para la Valoración Nutricional Antropométrica de la Niña y el Niño de 0 a 11 Años.

[19] Instituto Nacional de Salud (Perú) (2024). Tabla de Valoración Nutricional Antropométrica—Mujeres.

[20] Instituto Nacional de Salud (Perú) (2024). Tabla de Valoración Nutricional Antropométrica—Varones.

[21] Esteban-Figuerola P., Jardí C., Canals J., Arija V. (2020). Validación de un cuestionario corto de frecuencia de consumo alimentario en niños pequeños. Nutr. Hosp..

[22] Ministerio de Educación (MINEDU) (2016). Programa Curricular de Educación Primaria.

[23] World Medical Association (2013). World Medical Association Declaration of Helsinki: Ethical Principles for Medical Research Involving Human Subjects. J. Am. Med. Assoc..

[24] World Conference on Research Integrity (2010). Singapore Statement on Research Integrity.

[25] Kostecka M. (2022). The Effect of the “Colorful Eating Is Healthy Eating” Long-Term Nutrition Education Program for 3- to 6-Year-Olds on Eating Habits in the Family and Parental Nutrition Knowledge. Int. J. Environ. Res. Public Health.

[26] Yeh Y., Hartlieb K.B., Danford C., Catherine Jen K.L. (2018). Effectiveness of Nutrition Intervention in a Selected Group of Overweight and Obese African-American Preschoolers. J. Racial Ethn. Health Disparities.

[27] Qian L., Zhang F., Newman I.M., Shell D.F., Du W. (2017). Effects of selected socio-demographic characteristics on nutrition knowledge and eating behavior of elementary students in two provinces in China. BMC Public Health.

[28] Lim H., Kim J., Wang Y., Min J., Carvajal N.A., Lloyd C.W. (2016). Child health promotion program in South Korea in collaboration with US National Aeronautics and Space Administration: Improvement in dietary and nutrition knowledge of young children. Nutr. Res. Pract..

[29] Congreso de la República del Perú Ley N.° 30021: Ley de Promoción de la Alimentación Saludable para Niños, Niñas y Adolescentes. *Diario Oficial El Peruano*, 17 de mayo de 2013. https://www.gob.pe/institucion/congreso-de-la-republica/normas-legales/118470-30021.

[30] Ministerio de Salud (Perú) (2020). Resolución Ministerial N.° 033-2020-MINSA: Criterios de Evaluación a Quioscos, Cafeterías y Comedores Escolares en Instituciones de Educación Básica Regular Públicas y Privadas para una Alimentación Saludable.

