# Supplementary material for: Influence of a Nutrition Education Program on Parental Nutrition Knowledge, Dietary Habits, and Nutritional Status in Schoolchildren with Excess Weight
Source: Nutrients. 2026 Feb 13;18(4):613. doi: 10.3390/nu18040613 (PMC12942948; doi:10.3390/nu18040613)
Supplement: Supplementary file 1 [file nutrients-18-00613-s001.zip › File S1. Parental Nutritional Knowledge Questionnaire.docx]

**Food knowledge questionnaire for parents**

**PRE-POST TEST**

***Code: ______________________________________________***

***Your child's grade and section: _____________________Date: __________***

Instructions:

Good morning, parents, in coordination with the principal in charge of this educational institution, we are gathering information about your knowledge of topics related to healthy eating. We therefore ask for your sincere cooperation in filling out the following form. We thank you in advance for your participation.

**I. General Information:**

Educational Level: Preschool__ Elementary __ Middle School__ Technical__ Higher Education__

Have you previously received educational sessions on healthy eating topics? Yes __ No __

**II. Knowledge about healthy eating:**

The questions will be read to you and your answers will be marked with an X or circled by the evaluator.

1. Which food group protects your child against disease?

a) Builders

b) Regulators

c) Energy

2. Foods that help children grow, due to their building properties, are:

a) Oranges, star apples, rose apples, other fruits

b) Cassava, sweet potatoes, potatoes

c) Eggs, cheese, boiled chicken, other meats

3. The recommended number of meals per day for school-age children is:

a) 3

b) 4

c) 5

4. Mark true (T) or false (F) as appropriate:

a) We should wash our hands whenever necessary. ( )

b) Accessories should not be removed from the wrists when washing hands. ( )

c) Raw and cooked foods should not be placed in the same container. ( )

d) When cooking, we must keep utensils and workspaces clean. ( )

5. What is anemia?

a) A decrease in hemoglobin in the blood

b) It is an infection

c) A decrease in white blood cells

d) A decrease in cholesterol

6. What are the consequences of anemia and overweight in schoolchildren?

a) Schoolchildren tend to be pale, weak, fatigued, and have reduced learning capacity

b) Helps schoolchildren grow

c) Improves physical activity and school performance

7. How many times a week should you pack your child's lunch?

a) Every day

b) 5 times a week

c) 3 to 4 times a week

8. Which of the following examples is an appropriate lunchbox for schoolchildren?

a) Soft drink, bread, egg, fruit

b) Bread, hot dog, Frugos soft drink

c) Milk, water, steak sandwich, fruit, cookie

d) Yogurt, filled cookie, fruit, water

9. Mark true (T) or false (F) as appropriate:

a) Bone soup is a source of calcium ( )

b) Beets contain high amounts of iron ( )

c) A chubby child can have anemia ( )

d) Soy milk is nutritious for your child ( )

e) Fruit extracts provide vitamins for your child. ( )

10. Which of the following foods is considered iron-friendly because it helps iron enter the bloodstream and increase hemoglobin levels?

a) Herbal teas

b) Soda

c) Coffee

d) Orange juice, citrus fruit drinks

11. Which of the following plant-based foods contain the most iron?

a) Beetroot

b) Radish

c) Pumpkin

d) Lentils
