# Supplementary material for: Influence of a Nutrition Education Program on Parental Nutrition Knowledge, Dietary Habits, and Nutritional Status in Schoolchildren with Excess Weight
Source: Nutrients. 2026 Feb 13;18(4):613. doi: 10.3390/nu18040613 (PMC12942948; doi:10.3390/nu18040613)
Supplement: Supplementary file 1 [file nutrients-18-00613-s001.zip › File S2. Food Frequency Questionnaire (FFQ) – 69 Items.docx]

**Food consumption frequency questionnaire**

**PRE-POST TEST**

***Code: ______________________________________________***

***Your child's grade and section: _____________________Date: __________***

| **FOOD GROUP** | **No.** | **FOOD** | **Frequency of Consumption** | | | | |
| --- | --- | --- | --- | --- | --- | --- | --- |
|  |  |  | **Daily** | **4-6 weeks** | **1-3**  **weeks** | **1-3 months** | **never** |
| **GROUP 1 Cereals, tubers, and legumes** | **1** | **Rice** | ① | ② | ③ | ④ | ⑤ |
|  | **2** | **Banana flour** | ① | ② | ③ | ④ | ⑤ |
|  | **3** | **Noodles** | ① | ② | ③ | ④ | ⑤ |
|  | **4** | **Chiclayo Bean** | ① | ② | ③ | ④ | ⑤ |
|  | **5** | **Ucayalino beans** | ① | ② | ③ | ④ | ⑤ |
|  | **6** | **Bread** | ① | ② | ③ | ④ | ⑤ |
|  | **7** | **Potato** | ① | ② | ③ | ④ | ⑤ |
|  | **8** | **Sacha potato** | ① | ② | ③ | ④ | ⑤ |
|  | **9** | **Cassava** | ① | ② | ③ | ④ | ⑤ |
|  | **10** | **Banana** | ① | ② | ③ | ④ | ⑤ |
|  | **11** | **Toasted cassava flour** | ① | ② | ③ | ④ | ⑤ |
|  | **12** | **Tapioca** | ① | ② | ③ | ④ | ⑤ |
| **GROUP 2 Meat, fish, and eggs.** | **13** | **Canned tuna** | ① | ② | ③ | ④ | ⑤ |
|  | **14** | **Pork, Pulp** | ① | ② | ③ | ④ | ⑤ |
|  | **15** | **Heart (Chicken)** | ① | ② | ③ | ④ | ⑤ |
|  | **16** | **Liver (chicken)** | ① | ② | ③ | ④ | ⑤ |
|  | **17** | **Chicken egg** | ① | ② | ③ | ④ | ⑤ |
|  | **18** | **Fish** | ① | ② | ③ | ④ | ⑤ |
|  | **19** | **Chicken, Pulp** | ① | ② | ③ | ④ | ⑤ |
|  | **20** | **Beef, Pulp** | ① | ② | ③ | ④ | ⑤ |
|  | **21** | **Offal** | ① | ② | ③ | ④ | ⑤ |

| **FOOD GROUP** | **No.** | **FOOD** | **Frequency of Consumption** | | | | |
| --- | --- | --- | --- | --- | --- | --- | --- |
|  |  |  | **Daily** | **4-6 weeks** | **1-3**  **weeks** | **1-3 months** | **never** |
| **GROUP 3 Fruits** | **22** | **Aguaje** | ① | ② | ③ | ④ | ⑤ |
|  | **23** | **Caimito** | ① | ② | ③ | ④ | ⑤ |
|  | **24** | **Sugar cane** | ① | ② | ③ | ④ | ⑤ |
|  | **25** | **Carambola** | ① | ② | ③ | ④ | ⑤ |
|  | **26** | **Coconut** | ① | ② | ③ | ④ | ⑤ |
|  | **27** | **Cocona** | ① | ② | ③ | ④ | ⑤ |
|  | **28** | **Guaba** | ① | ② | ③ | ④ | ⑤ |
|  | **29** | **Mamey** | ① | ② | ③ | ④ | ⑤ |
|  | **30** | **Mandarin** | ① | ② | ③ | ④ | ⑤ |
|  | **31** | **Regional mango** | ① | ② | ③ | ④ | ⑤ |
|  | **32** | **Orange** | ① | ② | ③ | ④ | ⑤ |
|  | **33** | **Papaya** | ① | ② | ③ | ④ | ⑤ |
|  | **34** | **Pineapple** | ① | ② | ③ | ④ | ⑤ |
|  | **35** | **Silk banana** | ① | ② | ③ | ④ | ⑤ |
|  | **36** | **Sachamango** | ① | ② | ③ | ④ | ⑤ |
|  | **37** | **Cider** | ① | ② | ③ | ④ | ⑤ |
|  | **38** | **Uvilla** | ① | ② | ③ | ④ | ⑤ |
| **GROUP 4 Vegetables** | **39** | **Sweet pepper** | ① | ② | ③ | ④ | ⑤ |
|  | **40** | **Lettuce** | ① | ② | ③ | ④ | ⑤ |
|  | **41** | **Regional cucumber** | ① | ② | ③ | ④ | ⑤ |
|  | **42** | **Regional tomato** | ① | ② | ③ | ④ | ⑤ |
|  | **43** | **Caihua** | ① | ② | ③ | ④ | ⑤ |

| **FOOD GROUP** | **No.** | **FOOD** | **Frequency of Consumption** | | | | |
| --- | --- | --- | --- | --- | --- | --- | --- |
|  |  |  | **Daily** | **4-6 weeks** | **1-3**  **weeks** | **1-3 months** | **never** |
| **GROUP 5 Sugars and derivatives** | **44** | **Sugar (brown or white)** | ⑤ | ④ | ③ | ② | ① |
|  | **45** | **Honey** | ⑤ | ④ | ③ | ② | ① |
|  | **46** | **Chancaca** | ⑤ | ④ | ③ | ② | ① |
| **GROUP 6 Dairy products and derivatives** | **47** | **Evaporated milk** | ① | ② | ③ | ④ | ⑤ |
|  | **48** | **Fresh milk** | ① | ② | ③ | ④ | ⑤ |
|  | **49** | **Fresh cheese** | ① | ② | ③ | ④ | ⑤ |
| **GROUP 7 Fats** | **50** | **Vegetable oil** | ⑤ | ④ | ③ | ② | ① |
|  | **51** | **Coconut oil** | ⑤ | ④ | ③ | ② | ① |
|  | **52** | **Butter** | ⑤ | ④ | ③ | ② | ① |
|  | **53** | **Peanuts** | ⑤ | ④ | ③ | ② | ① |

| **FOOD GROUP**  **FOOD** | **No.** | **FOOD** | **Frequency of Consumption** | | | | |
| --- | --- | --- | --- | --- | --- | --- | --- |
|  |  |  | **Daily** | **4-6 weeks** | **1-3**  **weeks** | **1-3 months** | **never** |
| **GROUP 8 Prepared products** | **54** | **Natural Soft drinks** | ⑤ | ④ | ③ | ② | ① |
|  | **55** | **Soft drinks** | ⑤ | ④ | ③ | ② | ① |
|  | **56** | **Cookies** | ⑤ | ④ | ③ | ② | ① |
|  | **57** | **French fries, popcorn, snacks, cheese tris** | ⑤ | ④ | ③ | ② | ① |
|  | **58** | **Flan** | ⑤ | ④ | ③ | ② | ① |
|  | **59** | **Gelatin** | ⑤ | ④ | ③ | ② | ① |
|  | **60** | **Candy** | ⑤ | ④ | ③ | ② | ① |
|  | **61** | **Jams** | ⑤ | ④ | ③ | ② | ① |
|  | **62** | **Hot dog** | ⑤ | ④ | ③ | ② | ① |
|  | **63** | **White dessert** | ⑤ | ④ | ③ | ② | ① |
|  | **64** | **Grill chorizo** | ⑤ | ④ | ③ | ② | ① |
|  | **65** | **Salchipapas** | ⑤ | ④ | ③ | ② | ① |
|  | **66** | **Broster (Fried)** | ⑤ | ④ | ③ | ② | ① |
|  | **67** | **Cakes, pies, desserts** | ⑤ | ④ | ③ | ② | ① |
|  | **68** | **Bottled juices (Frugos, etc.)** | ⑤ | ④ | ③ | ② | ① |
|  | **69** | **Grilled Chicken** | ⑤ | ④ | ③ | ② | ① |
