# Supplementary material for: Influence of a Nutrition Education Program on Parental Nutrition Knowledge, Dietary Habits, and Nutritional Status in Schoolchildren with Excess Weight
Source: Nutrients. 2026 Feb 13;18(4):613. doi: 10.3390/nu18040613 (PMC12942948; doi:10.3390/nu18040613)
Supplement: Supplementary file 1 [file nutrients-18-00613-s001.zip › Table S1. Post-hoc pairwise comparisons of parental nutritional knowledge scores by group and time.docx]

| Table S1. Post-hoc pairwise comparisons of parental nutritional knowledge scores by group and time | | | | |
| --- | --- | --- | --- | --- |
| **Group** | **Comparison** | **Mean difference (Post–Pre)** | **95% CI** | **Bonferroni p-value** |
| Experimental | Post vs Pre | +4.50 | 2.10 - 6.90 | 0.001 |
| Control | Post vs Pre | −1.15 | −3.20 - 0.90 | 0.28 |
| Values correspond to estimated marginal means derived from linear mixed-effects models. CI: 95% confidence interval. | | | | |
